# Immune repertoire analysis of normal Chinese donors at different ages

**DOI:** 10.1111/cpr.13311

**Published:** 2022-08-04

**Authors:** Cailing Song, Wenjing Pan, Brittany Brown, Congli Tang, Yunqi Huang, Houao Chen, Nan Peng, Zhe Wang, Daniel Weber, Miranda Byrne‐Steele, Haijing Wu, Hongna Liu, Yan Deng, Nongyue He, Song Li

**Affiliations:** ^1^ Hunan Key Laboratory of Biomedical Nanomaterials and Devices Hunan University of Technology Zhuzhou China; ^2^ Nanjing ARP Biotechnology Co., Ltd. Nanjing China; ^3^ iRepertoire Inc. Huntsville Alabama USA; ^4^ Guangdong Provincial Hospital of Chinese Medicine & Guangdong Provincial Academy of Chinese Medical Sciences Guangzhou China; ^5^ Department of Dermatology, Second Xiangya Hospital, Hunan Key Laboratory of Medical Epigenomics Central South University Changsha China

## Abstract

**Objectives:**

This study investigated the characteristics of the immune repertoire in normal Chinese individuals of different ages.

**Materials and Methods:**

In this study, all seven receptor chains from both B and T cells in peripheral blood of 16 normal Chinese individuals from two age groups were analyzed using high‐throughput sequencing and dimer‐avoided multiplex PCR amplification. Normal in this study is defined as no chronic, infectious or autoimmune disease within 6 months prior to blood draw.

**Results:**

We found that compared with the younger group, the clonal expression of T‐cell receptor repertoire increased in the older group, while diversity decreased. In addition, we found that the T‐cell receptor repertoire was more significantly affected by age than the B‐cell receptor repertoire, including significant differences in the use of the unique TCR‐alpha and TCR‐beta V‐J gene combinations, in the two groups of normal participants. We further analyzed the degree of complementarity determining region 3 sequence sharing between the two groups, and found shared TCR‐alpha, TCR‐gamma, immunoglobulin‐kappa and immunoglobulin‐lambda chain complementarity determining region 3 sequences in all subjects.

**Conclusion:**

Taken together, our study gives us a better understanding of the immune repertoire of different normal Chinese people, and these results can be applied to the treatment of age‐related diseases. Immune repertoire analysis also allows us to observe participant's wellness, aiding in early‐stage diagnosis.

## Graphical abstract

With aging, the body's thymus involution, T and B cells senescence, T cell clones expand, the diversity of the TCR repertoire decreases, and the immunity of the body's immune system decreases.

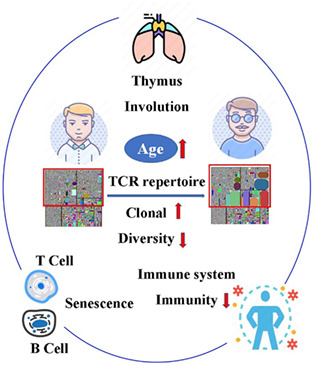

## 1 INTRODUCTION

The Adaptome is a collection of all the structural and functional diversity of T cell receptors (TCRs) and B cell receptors (BCRs) of an individual at any given time, which accurately and comprehensively reflects the dynamic changes of the immune system.^1^ Within the adaptome, the repertoire consists of all seven chains from TCR and BCRs and their clonotypes.^2^, ^3^, ^4^ Immune repertoire high throughput sequencing (IR‐HTS) is the study of the full‐length receptor or complementarity determining region 3 (CDR3) region from both TCRs and BCRs to assess immune system diversity and immune system relationship to disease.^5^ Each peptide chain of TCR and BCR includes a variable (V), diversity (D), joining (J) and constant (C) region.^6^ The V region includes three CDRs (CDR1, CDR2, and CDR3) with CDR3 having the greatest variability.^7^ IR diversity is due to the V (D) J gene rearrangement and addition or deletion of nucleotides in the CDR3 region, as well as somatic hypermutation (SHM) in B cells.^8^, ^9^ With the rapid development of HTS technology, it is increasingly applied to complex structures and large‐scale IR analysis.^10^, ^11^ An accurate and comprehensive understanding of the diversity of TCRs and BCRs in different age groups will facilitate the development of new immunotherapy measures for diseases^12^, ^13^ and the provision of personalized therapies for patients.^14^, ^15^, ^16^

In recent years, with the rapid development of HTS technology, IR has been widely used in disease‐related research.^17^, ^18^, ^19^, ^20^, ^21^, ^22^, ^23^ One study found that there were differences in the use of V and J genes in both β and IGH chains in patients with non‐small cell lung cancer (NSCLC). It was found that the clonality and diversity of the TCR repertoire in the early recurrence patients were increased and decreased, respectively, compared to the early non‐recurrence patients.^24^ Wang's team used HTS to study TCR diversity in the peripheral blood and bone marrow of patients with acute lymphoblastic leukaemia before and after treatment, and found that the diversity of TCRs in peripheral blood and bone marrow decreased and the clonality increased after treatment with chimeric antigen receptor T cells (CAR‐T).^25^ One study reported a significant increase in TCR β chain CDR3 diversity in normal participants after the second hepatitis B vaccination, while a significant decrease in BCR IgG Heavy chain CDR3 diversity.^26^ Another study shows in patients with coronavirus disease 2019 (COVID‐19), the TCR repertoire diversity was reduced, and the TCR β clone is expanded. This study also showed that the CDR3 sequence length of TCR is longer in COVID‐19 patients that were co‐infected with pneumonia compared with patients without pneumonia.^27^ The above studies indicate that the diversity of IR is related to the occurrence and development of diseases.^28^, ^29^, ^30^ At the same time, the above studies have also enabled us to have a better understanding of the IR, but our understanding of the IR of normal Chinese people of different ages is still very limited. Therefore, the study of IR in normal Chinese subjects of different ages has tremendous implications for the treatment of age‐related diseases.^31^, ^32^, ^33^, ^34^

Dimer‐avoided multiplex PCR (Dam‐PCR) can be used to amplify all seven chains of the IR in the same PCR reaction system under the same conditions, with unique molecular IDs (UMIs), which can reduce the amplification bias and directly compare the gene expression level quantitatively. In this study, 16 normal Chinese participants were divided into two groups based on age: Group 1 (ages 19–30) and Group 2 (ages 50–67), with eight participants in each group. Peripheral blood samples from the participants were collected, RNA was extracted and Dam‐PCR combined with HTS was used to analyze the IR of each participant. The results showed that with the increase of age, the diversity of peripheral blood TCR repertoire decreased, and the average length of TRA CDR3 sequence increased significantly. In addition, we found that V and J genes existed independently in both groups, and the expression difference of TRB V‐J gene combination between the two groups was the most significant. Shared CDR3 sequences from TRA, TRG, IGK, and IGL were found among all individuals. Our results were designed to reveal the diversity of the IR in Chinese normal subjects of different ages.

## 2 MATERIALS AND METHODS

### 2.1 Sample collection

Sixteen normal participants were included in the study. The experiment was divided into two groups: Group 1, ages 19–30, and Group 2, ages 50–67, with eight participants in each age group. The participants were excluded from neurological, infectious, autoimmune and other serious cardiovascular and cerebrovascular diseases. Peripheral blood samples were collected from participants using BD PAXgene Blood RNA Tube (Ca# 762165, BD, US). The study was approved by the NanJing XiaoZhuang University Ethics Committee (lot number 2021NO.01) and the informed consent of all normal participants was obtained.

### 2.2 RNA extraction

The PAXgene Blood RNA Kit (Ca# 762174, PreAnalytix, US) was used to extract RNA from peripheral blood from all 16 participants. The concentration and purity of RNA were measured by NanoDrop One UV spectrophotometer.

### 2.3 IR amplification and sequencing

iRepertoire's automated iR‐RepSeq‐plus 7‐Chain Cassette (Ca# iR + 7chain‐HLRI‐C, iRepertoire, US) was used to construct a 7‐chain library for each sample. Each cassette contains all reagents needed to perform Dam‐PCR. 1200 ng of RNA was used as template per cassette. The iR‐RepSeq‐plus cassette performs four rounds of PCR: first strand synthesis (FSS), second strand synthesis (SSS), and two rounds of enrichment. FSS contains a gene specific reverse primer mix for reverse transcription and first strand cDNA synthesis. Following FSS, unused primers are removed and a gene specific forward primer mix performs second strand cDNA synthesis. Residual primers are removed after SSS and a 2‐step enrichment PCR with Illumina communal adaptor sequences is performed for amplification and enrichment. Detailed library construction procedures are available in the literature^35^ and on iRepertoire's website. SPRIselect magnetic beads were used to clean the libraries. Each sample was individually barcoded, pooled and multiplexed in one sequencing run and de‐multiplexed in downstream data analysis. The multiplexed library was sequenced on a 500‐cycle kit on the Illumina NovaSeq platform.

### 2.4 Statistical analysis

The iRmap program was used to analyze the raw data.^36^ GraphPad Prism 8.0 was used for statistical analysis. NS indicates no statistical difference, *p < 0.05 indicated a statistical difference, while **p < 0.01 indicated a significant difference.

## 3 RESULTS

### 3.1 Research participant metadata

The purpose of this study was to study the characteristics of all seven chains from peripheral blood IR in Chinese normal subjects of different ages. Sixteen normal participants were coded from 1 to 16. Table 1 describes participant data from the 16 normal participants and sequencing results of seven chains. By analyzing the total RNA receptor count and the unique CDR3 (uCDR3) obtained by sequencing, we found that total receptor RNA in Group 1 averaged about 180,000 total RNA and an average of 60,000 total uCDR3. In Group 2, total receptor RNA averaged about 240,000 and total uCDR3 about 80,000. uCDR3 discovery for both groups were similar, around 30% uCDR3 from total reads. The above results show that the sequencing results of all samples are successful and can be used for subsequent analysis.

**TABLE 1:** Research participant metadata and sequencing results

Sample ID	Sex (M/F)	Race	Ethnicity	Age	Age group	Total receptor RNA	Total uCDR3
1	F	Asian	Han	20	19–30	176735	72300
2	F	Asian	Han	22		316526	91389
3	M	Asian	Han	27		110693	53826
4	F	Asian	Han	29		139996	79333
5	F	Asian	Han	30		272024	79185
6	F	Asian	Han	24		112408	27594
7	M	Asian	Han	20		160304	64425
8	F	Asian	Han	19		208671	69678
9	F	Asian	Han	50		416159	119173
10	F	Asian	Han	52	50–67	211901	74161
11	M	Asian	Han	60		184716	79518
12	F	Asian	Han	60		275400	114450
13	M	Asian	Han	60		379226	91829
14	F	Asian	Han	60		89974	38232
15	M	Asian	Han	65		192929	68031
16	F	Asian	Han	67		193569	88172

### 3.2 Adaptome analysis

**FIGURE 1:**
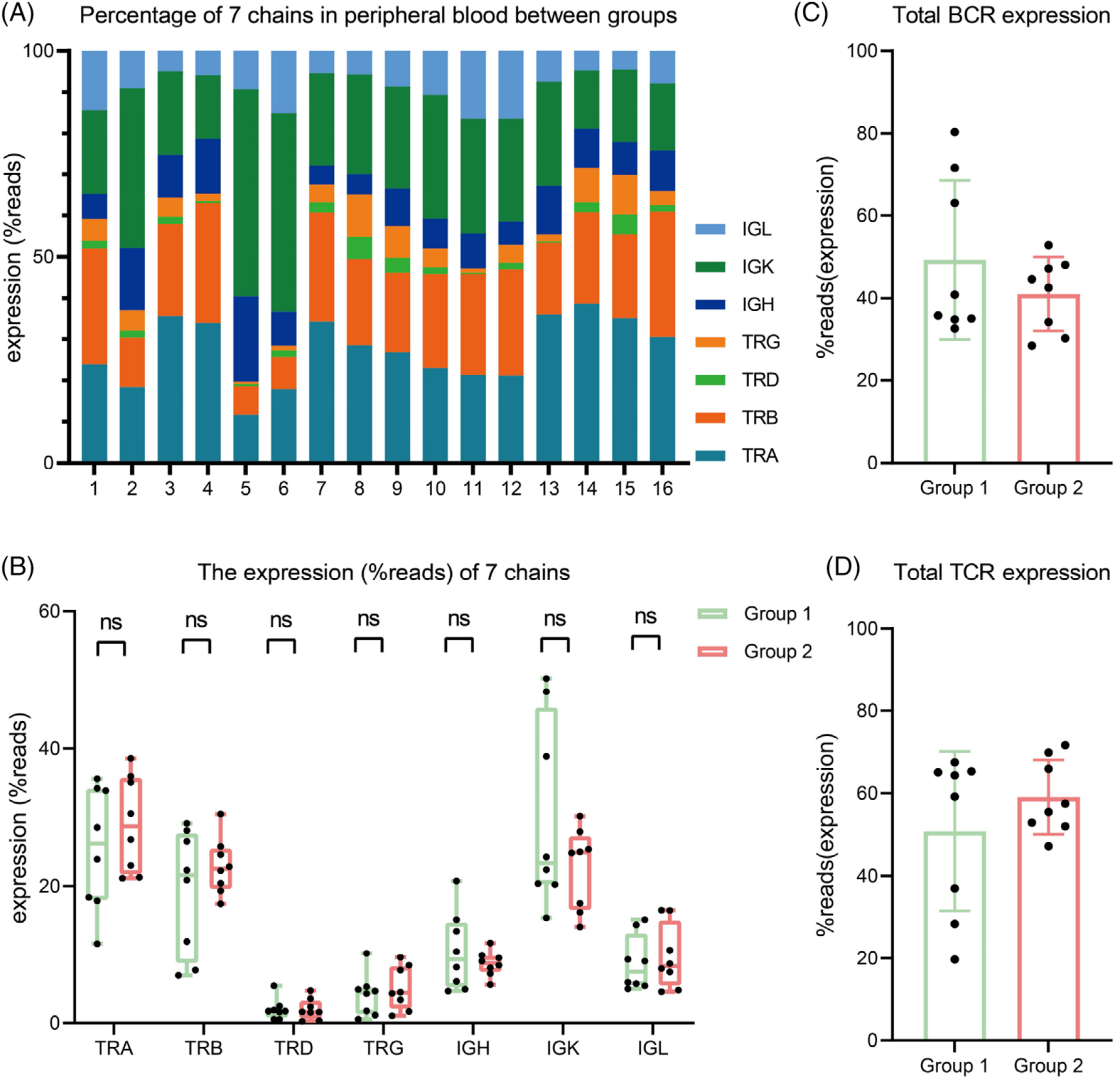
Seven chains IR analysis between groups. (A) Percentage of seven chains in peripheral blood between groups. Each column represents a study subject, numbered 1–8 for Group 1 and numbered 9–16 for Group 2. The same color block represents the same chain and its size represents the percentage of this chain expression. (B) Comparison of seven chains expression percentages between groups. (C) BCR percentage between groups. (D) TCR percentage between groups. (B), (C) and (D) were analyzed by *t* test. Mean ± SEM was shown. ns indicates no statistical difference.

Adaptome refers to all B and T cell receptor genes expressed in a sample. Differences were found in the seven chains repertoire expression between the two groups (Figure 1A). It can be seen that the TCR and BCR repertoires each accounted for about half of the IR in Group 1, while TCR repertoire expression increased in Group 2 (Figure 1C,D). It was also found in both groups that the expression levels of TRA and TRB were significantly higher than TRD and TRG, while IGK expression levels were significantly higher than IGH and IGL. The mean expression levels of TRA, TRB, IGK, and IGL in Group 2 were higher compared with Group 1, while the mean expression level of IGH was slightly lower (Figure 1B).

### 3.3 CDR3 region sequence length analysis

**FIGURE 2:**
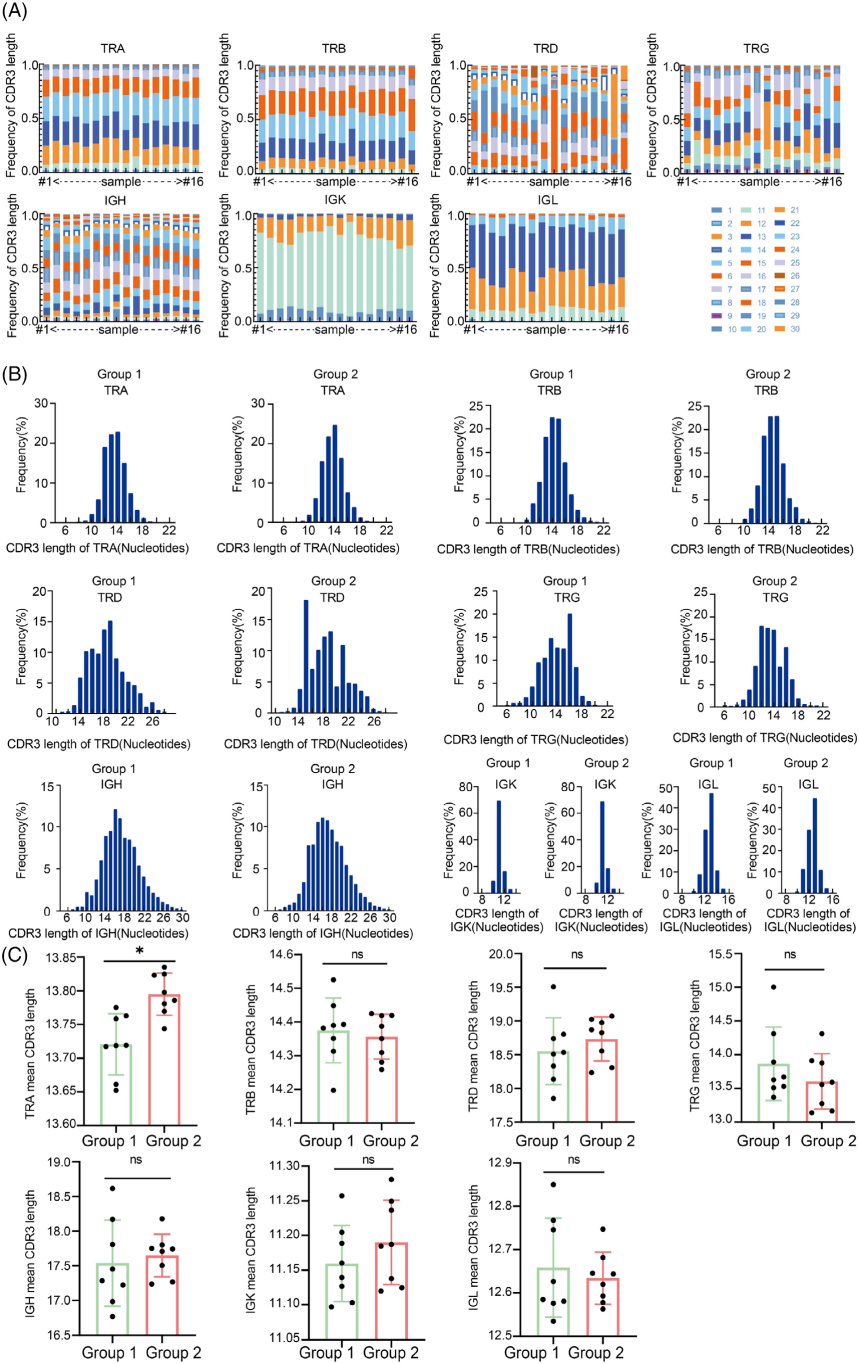
Distribution characteristics of the CDR3 region sequence length in IR from peripheral blood. (A) Seven chains CDR3 sequence length distribution of IR in each participant. Each column represents a study subject, #1 is the numbered 1 subject, #16 is the numbered 16 subject, from left to right is 1–16. The same color block represents the same length of CDR3, and its size represents the frequency. (B) Seven chains CDR3 region sequence length distribution between groups. (C) Comparison of mean sequence length of seven chains CDR3 region between groups of normal participants. (C) was analyzed by *t* test. Mean ± SEM was shown. ns indicates no statistical difference and * represents a statistical difference.

The CDR3 is of particular interest because it is the most variable portion of the antigen‐biding site, and is associated with antigen‐specificity. The longer the length of CDR3, the more complex the peptide chain folding structure and the higher the specificity of the receptor. CDR3 length from both T and B lymphocytes was compared between groups. TCR and BCR CDR3 regions had the greatest variability within the seven chains repertoire. Significant differences in CDR3 nucleotide length were found among the participants (Figure 2A). It was found that the morphology of the CDR3 region sequence length distribution maps of TRB, IGK, and IGL chains in the two groups of normal participants was similar (Figure 2B). However, the sequence length distribution maps of CDR3 region of TRD and TRG showed significant morphological differences (Figure 2B). Further analysis of the average CDR3 sequence length showed that with an increase in age, the average CDR3 sequence length of TRA, TRD, IGH, and IGK increased significantly, while the average CDR3 sequence length of TRB, TRG, and IGL decreased, but there was no statistical significance (Figure 2C).

### 3.4 Treemap and D50 analysis

**FIGURE 3:**
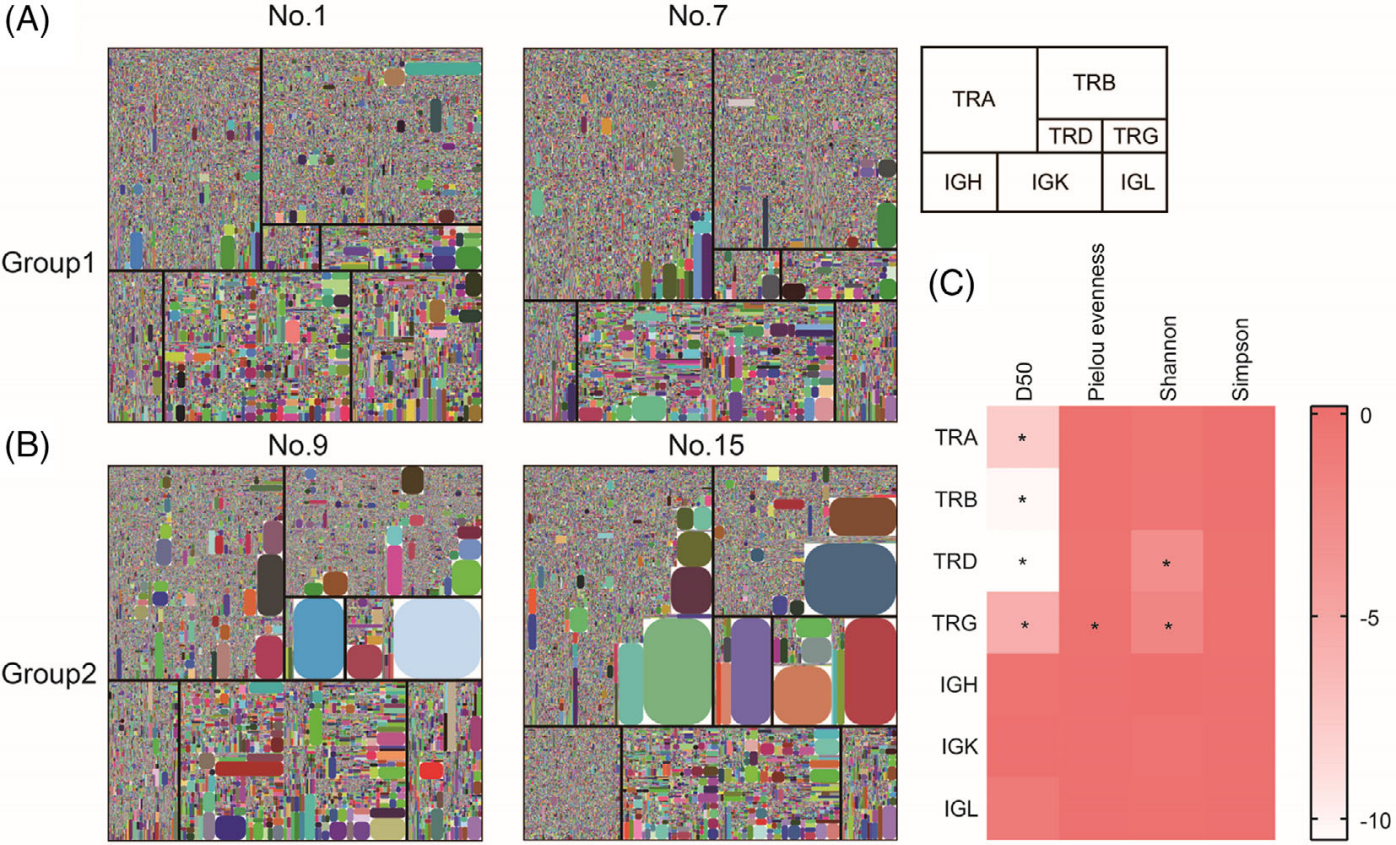
IR diversity of the two groups of normal participants. (A) Treemap of two individual participants from Group 1. (B) Treemap of two individual participants from Group 2. No. 1, No. 7, No. 9, No. 15 in (A) and (B) represent normal participant with the numbers 1, 7, 9 and 15, respectively. (C) Comparison of diversity indexes of seven chains between the two groups. Each column represents a diversity indicator, and one cell in each row represents the difference between the average value of Group 2 index and Group 1 Index (the lowest is white and the highest is red).

Treemap, developed by iRepertoire, can visually describe the diversity of immune repertoire in a given sample. The Treemap reads from left to right and top to bottom as TRA, TRB, TRD, TRG, IGH, IGK, and IGL. Each rounded rectangle represents a unique CDR3, where the size of the spot represents the relative frequency. The different sizes are related to clonal expansion within the immune repertoire. The more uCDR3, or spots, generally represents a higher diversity in the given immune repertoire. Treemaps from two individuals in Group 1 and two individuals in Group 2, can be seen in Figure 3A,B, respectively. As can be seen from Figure 3A,B, TRA, TRB, and IGK occupied a large area in the IR of both groups. Compared with Group 2, the color blocks in the TCR Treemap of Group 1 contained less clonal expansion, thus having a higher diversity.

D50 is a numerical value developed by iRepertoire to quantitatively describe the diversity of the immune database. D50 is the percentage of the total number of unique CDR3 that account for the cumulative 50% of the total CDR3s in a given sample, and the closer it is to 50, the more diverse a library. Shannon, Simpson, and Pielou evenness indexes were also used to describe the diversity and evenness of the IR. The diversity index analysis of the seven chains in the IR of the two groups is shown in Figure 3C. As can be seen from Figure 3C, compared with Group 1, the TCR D50 values in Group 2 were significantly reduced. The D50 values of the BCR repertoire between the groups were not significantly different. Shannon index of TRD in Group 2 showed a significant decrease compared to Group 1. Both Shannon and Pielou evenness indices showed a significant decrease in TRD and TRG diversity and homogeneity in Group 2.

### 3.5 V and J gene analysis

**FIGURE 4:**
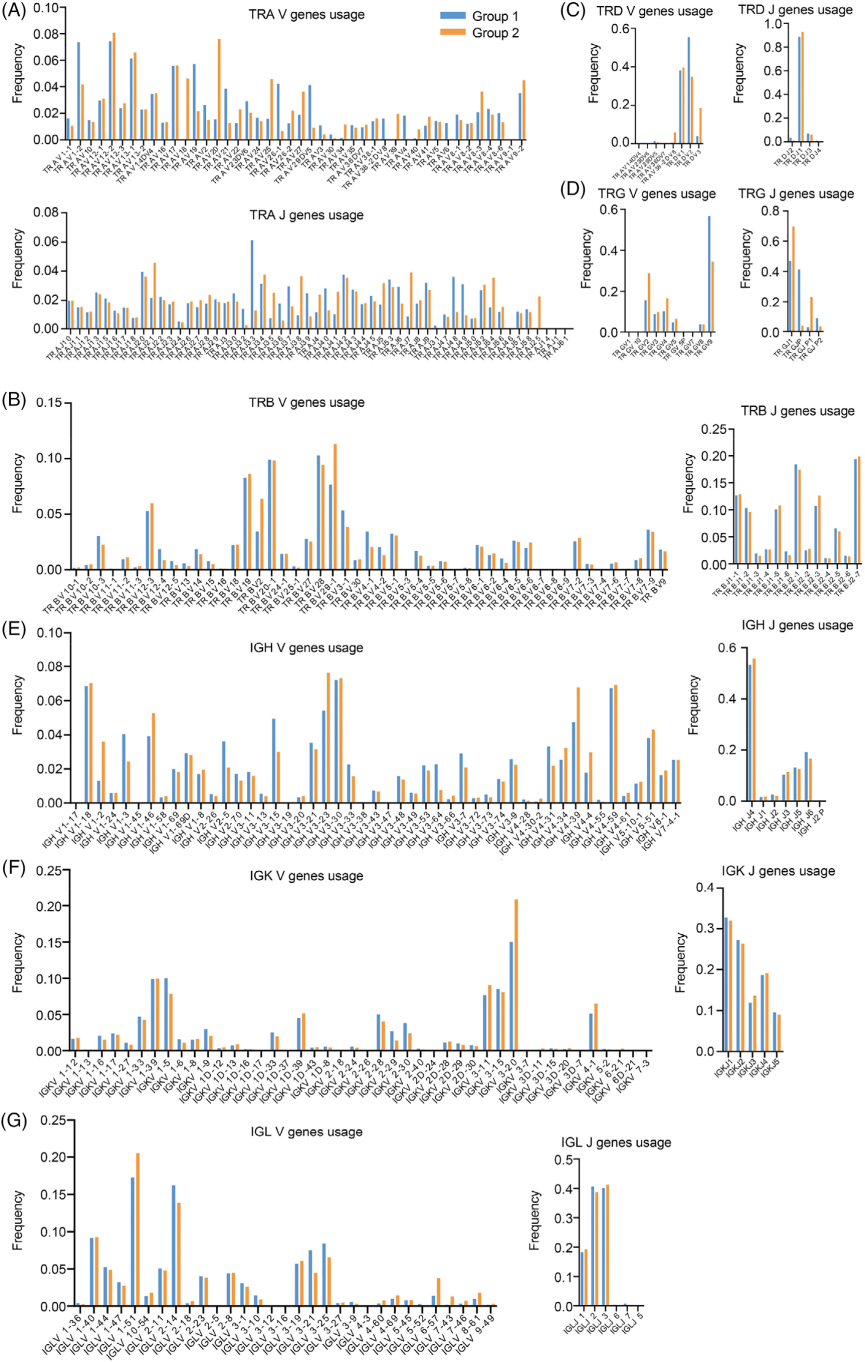
Seven chains V and J genes usage between groups. (A) TRA V and J genes usage between groups. (B) TRB V and J genes usage between groups. (C) TRD V and J genes usage between groups. (D) TRG V and J genes usage between groups. (E) IGH V and J genes usage between groups. (F) IGK V and J genes usage between groups. (G) IGL V and J genes usage between groups. Blue represents the Group 1, yellow represents the Group 2, the horizontal coordinate is the V/J gene type, and the vertical coordinate represents the V/J gene frequency.

The diversity of both TCR and BCR CDR3 regions is caused by recombination of V(D)J genes and addition or deletion of nucleotides. The CDR3 region was amplified and sequenced for both TCRs and BCRs to reveal the use of V and J genes in all seven chains. GraphPad Prism software was used to make statistics on the use frequency data of V and J genes and draw a 2‐dimensional histogram for comparison. Figure 4 shows the expression of V and J genes of all TCR and BCR chains in different age groups. Although most V/J genes were expressed similarly between the two groups, significant differences were found in the use of certain V/J genes. Overall, most of the genes that were high expressed in Group 1 were also observed in the Group 2. TRA was the only chain that showed a significant difference in gene expression between groups (Figure 4A). Within Group 2, significant increases were discovered in TRAV18, TRAV20, TRAJ21, TRAJ38, TRAJ54, and TRAJ25, while significant decreases were discovered in TRAV1‐2, TRAV19, TRAV21, TRAJ33, TRAJ48, and TRAJ49 genes (Figure 4A). Surprisingly, TRA V/J gene use differed the most between the two groups of normal participants (17 TRA V genes and 18 TRA J genes were expressed differently), with some genes being found only in Group 2, such as TRAV18, TRAV39, and TRAJ25. TRAV38‐2DV8, TRAV4, and TRAV6 genes were found only in Group 1 (Figure 4A). These results can indicate that the increase of age has a great influence on TRA gene use in normal Chinese people.

### 3.6 V‐J gene usage

**FIGURE 5:**
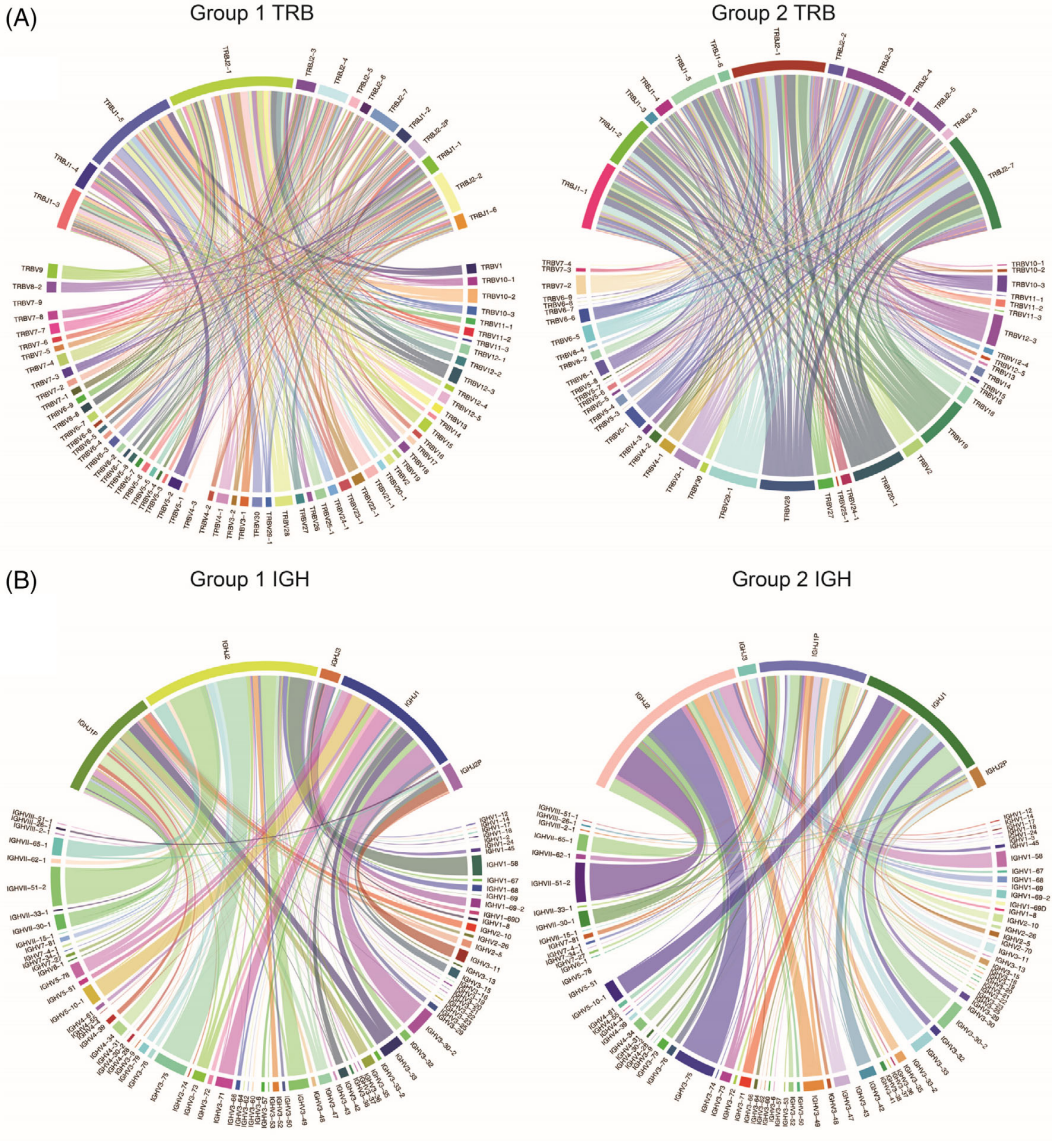
V‐J gene usage. (A) V‐J gene usage of TRB between the two groups. (B) V‐J gene usage of IGH between the two groups. Different colored arcs in the outer circle represent V/J gene types, and the size of the arc represents the frequency. The area of the inner circle V‐J gene linkage represents the combined V‐J gene expression frequency. The longer the outer circle arc and the larger the area of the inner circle V‐J gene linkage represents the higher frequency of V/J gene and V‐J gene combination use.

In order to observe the use of V‐J gene combination of seven chain receptors of the two groups, we amplified and sequenced the CDR3 region from all seven chains, and visually displayed the frequency of V‐J gene usage using 2D map. The results show that the TRA, TRD, TRG, IGH, IGK, and IGL V‐J gene combinations were similarly expressed in both groups (Figure 5B and Supplementary material Figure S1A–E). TRAV1‐2/TRAJ33, TRDV36DV7/TRDJ3, TRGV1/TRGJP2, IGHV3‐75/IGHJ2, IGKV3‐34/IGKJ4, and IGLV3‐16/IGLJ2 gene combinations were expressed the most of the two groups. These results suggest that the highly expressed combination of V‐J genes may play an important role in human health. In addition to the highly expressed TRA V/J genes, significant differences were also found in TRB V‐J gene combination expression between the two groups (Figure 5A). TRBV5‐2/TRBJ1‐5, TRBV10‐2/TRBJ1‐5, TRBV21‐1/TRBJ2‐1, and TRBV30/TRBJ1‐5 were highly expressed in Group 1. TRBV28/TRBJ2‐7, TRBV20‐1/TRBJ2‐7, TRBV19/TRBJ2‐1, TRBV29‐1/TRBJ2‐7, and TRBV20‐1/TRBJ2‐1 were highly expressed in Group 2, while the expression of TRBV5‐2/TRBJ1‐5, TRBV10‐2/TRBJ1‐5, TRBV21‐1/TRBJ2‐1, and TRBV30/TRBJ1‐5 decreased. These results indicated that the expression of TRB V‐J gene combination was different in Chinese normal subjects of different ages.

### 3.7 CDR3 sharing

**FIGURE 6:**
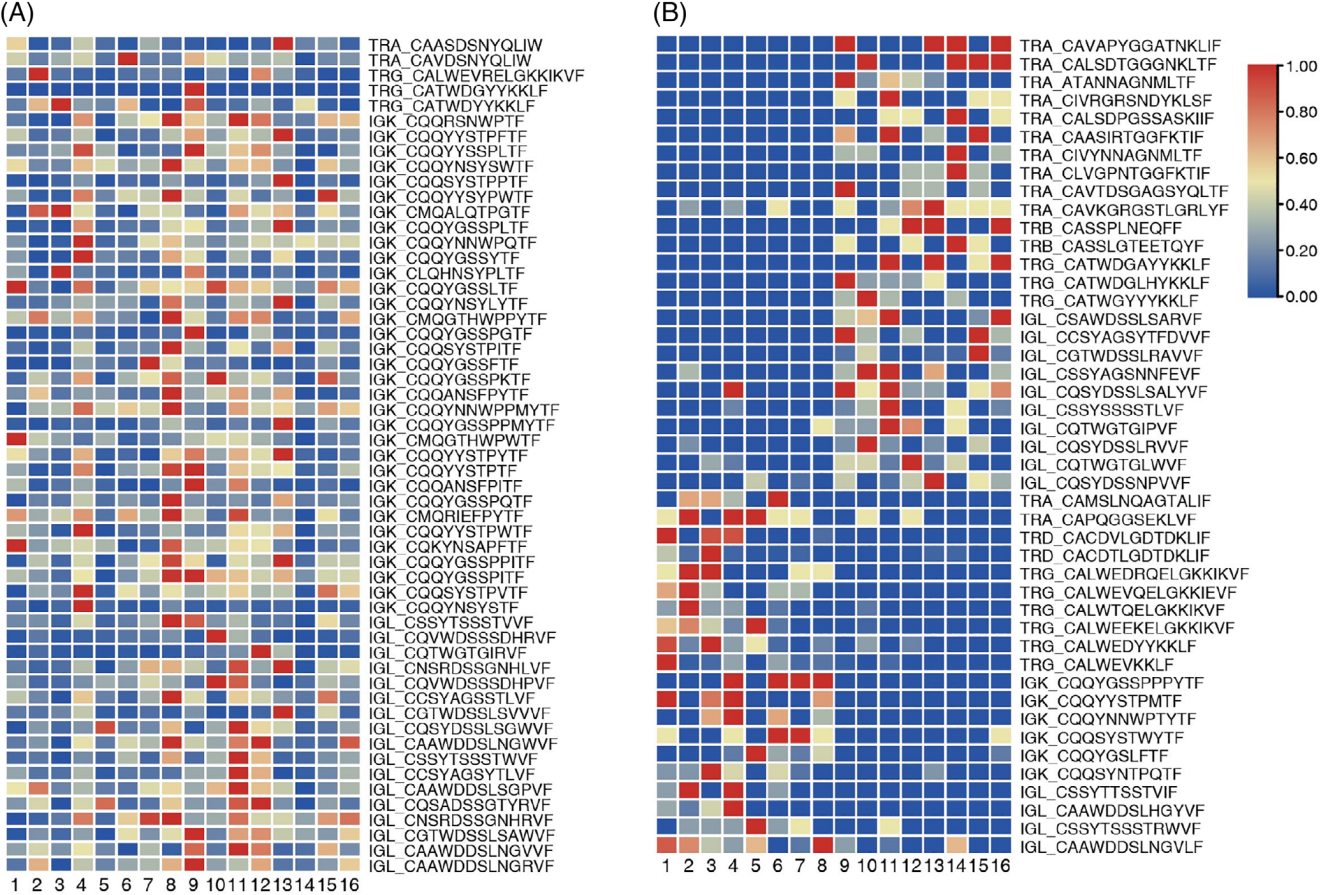
CDR3 sharing. (A) CDR3 sharing among participants. (B) Highly expressed CDR3s within groups.

To assess the prevalence of CDR3 sharing among both groups, we combined all sequencing data from the participants for analysis. It was found that almost all CDR3 sequences were unique in each participant, and only a few CDR3 sequences were shared among all participants (Figure 6A). Shared seven chain CDR3 sequences are expressed at different levels between groups. Using the Mann–Whitney *U* test, the expression level of uCDR3 sequences and representative sequences were plotted, showing significantly different expression between younger and older subjects (*p* < 0.05) (Figure 6B and Supplementary material S2). Each column represents an object, and a cell in each row represents the expression frequency of the CDR, (lowest as blue, highest as red).

We failed to detect TRB, TRD, and IGH CDR3 sequences shared among all participants in our study. However, we found shared TRA, TRG, IGK, and IGL CDR3 sequences in all participants (Figure 6A). Interestingly, we found a high number of TRA and IGK CDR3 sequences (100 and 274 sequences, respectively) shared among all participants (Figure 6A). Some shared sequences were highly expressed in all participants, such as CATWDGYYKKLF and CATWDYYKKLF (Figure 6A). These sequences are highly similar and may have similar functions, suggesting that these sequences may be related to maintaining human life and health. In addition, we found that older normal participants shared more TRA and IGL CDR3 sequences, while younger normal subjects shared more TRG and IGK CDR3 sequences (Figure 6B and Supplementary material S2).

## 4 DISCUSSION

Aging plays a big factor on the immune system.^37^, ^38^ As age increases, the potential ability of the immune system to resist newly encountered pathogens decreases, resulting in a decreased vaccination effect and an increased risk of age‐related diseases such as cancer, osteoporosis and diabetes.^39^, ^40^, ^41^, ^42^, ^43^, ^44^ The reduced naïve T‐cell repertoire in elderly COVID‐19 patients significantly reduces the body's ability to mount an adaptive immune response to the neoantigen SARS‐CoV‐2.^45^ Thevarajan et al. found rapid production and significant increase in activated CD8+ T cells in young COVID‐19 patients with good prognosis on day 7–9 after SARS‐CoV‐2 infection,^46^ while the number of CD4+ and CD8+ T cells was significantly reduced in elderly and critically ill patients.^47^ The above study showed a positive correlation between total T cells, CD4+ and CD8+ T cells and patient survival. It has been reported that the diversity of TRA and TRB in the elderly is lower than that in the young,^48^ our findings are consistent with this. However, it is still unclear how age affects the state of the immune system and the characteristics of the IR of normal individuals of different ages. This study allowed us to comprehensively analyse the full seven chains receptor from B and T cells in Chinese normal participants by using HTS and dam‐PCR amplification technology. Our results showed that the clonal expression of the TCR repertoire increased in Group 2, the older population, while the diversity of TCR repertoire decreased, suggesting that the increase of age may lead to the clonal expansion of some autoimmune T cells. TCR repertoire diversity was significantly different between different age groups and the degree of immune repertoire diversity is not dependent on gender.^49^

The recombination of V (D) J gene and the addition or deletion of nucleotides in CDR3 region leads to the diversity of the IR. Our study showed that there were differences in V and J gene use in different age groups, and the increase of age had a significant impact on TRA and TRB. We observed that TRAV18, TRAV39, and TRAJ25 genes were uniquely expressed in Group 2, the older population, while TRAV38‐2DV8, TRAV4, and TRAV6 genes were independently expressed in the Group 1, the younger population. At the same time, we observed significant differences in the expression of TRB V‐J gene combinations between the two groups of normal participants. The V‐J gene combination that was highly expressed in Group 1 was decreased in Group 2. These results showed that there may be a correlation between the expression of V and J genes and age. In addition, we observed some highly expressed shared CDR3 sequences in TRA, TRG, IGK, and IGL of all participants. T and B cells achieve antigen specificity through V(D)J gene rearrangement to produce more efficient and accurate clonal amplification, which is essential for a successful immune response.^1^ Peng et al. found increased use of some V gene segments and V‐J gene segment pairings in PBMC from Avian Influenza Virus Infection (AIV) donors compared to healthy donors, such as TRBV12‐3 and TRBV30/TRBJ1‐2, which could provide additional information for developing more effective treatment measures.^50^ Lian et al. found that TRB CDR3 amino acid sequences were most highly expressed between 21 and 30 years of age, and our results showed the presence of highly expressed shared TRB CDR3 amino acid sequences between Group 2, which were largely unexpressed in Group 1 (Figure 6B).^49^ We constructed CDR3 sequence models of the two age groups and can use this to compare CDR3 sequence characteristics of patients with corresponding ages, and how differentially expressed CDR3 sequences might be associated with disease. Heather et al. found that public TCR sequences were absent in HIV patients compared to healthy individuals, and that patients undergoing short‐term antiretroviral therapy were accompanied by rapid changes in the abundance of many individual TCR sequences, a reduction in the abundance of the most common sequences, and a reduction in most HIV‐associated CDR3 sequences.^51^

Our study shows that the IR of normal Chinese participants of different ages is individual specific. Our results provide us with a better understanding of the characteristics of the immune pool in normal Chinese people and a method which provides a foundation to study age‐related diseases and monitor treatments, thus improving future treatments. This study shows the importance of conducting larger studies of repertoire analysis from different ages, populations, and health status, as this can be used as disease markers thus aiding in early diagnosis^52^, ^53^, ^54^, ^55^ and personalized treatment.^56^, ^57^, ^58^, ^59^, ^60^

## AUTHOR CONTRIBUTIONS

Cailing Song performed the sample preparation, data analysis and wrote the manuscript. Wenjing Pan analyzed the data and revised the manuscript for important intellectual content. Houao Chen and Nan Peng collected the data and assisted in the data analysis. Congli Tang and Yunqi Huang assisted in the nucleic acid extraction and library construction. Brittany Brown, Zhe Wang, Daniel Weber, and Miranda Byrne‐Steele contributed reagents and analysis tools, Haijing Wu, Hongna Liu, Yan Deng, and Nongyue He provided revisions. Song Li initiated the project research, designed the experimental protocol, revised the manuscript, and provided funding. All authors have read and approved the manuscript for publication.

## CONFLICT OF INTEREST

Author Wenjing Pan and Congli Tang are employed by Nanjing ARP Biotechnology Co. Ltd, Nanjing, China. Author Brittany Brown, Daniel Weber, Miranda Byrne‐Steele are employed by iRepertoire Inc., Huntsville, USA. The remaining authors declare there is no conflict of interest.

## Supporting information


**FIGURE S1** V‐J gene usage. (A) V‐J gene usage of TRA between the two groups. (B) V‐J gene usage of TRD between the two groups. (C) V‐J gene usage of TRG between the two groups. (D) V‐J gene usage of IGK between the two groups. (E) V‐J gene usage of IGL between the two groups. Different colored arcs in the outer circle represent V/J gene types, and the size of the arc represents the frequency. The area of the inner circle V‐J gene linkage represents the combined V‐J gene expression frequency.

**APPENDIX S1** Supporting Information

## Data Availability

The dataset used in this study has been uploaded to the online repository: https://ngdc.cncb.ac.cn/gsa-human/, HRA002445. Further enquiries for all data and figures used in this study can be made to the corresponding authors.

## References

[1] Han J , Lotze MT . Adaptive immunity and the tumor microenvironment. In: Lee PP , Marincola FM , eds. Tumor Microenvironment. 1st ed. Springer Nature Switzerland AG; 2020:112‐134.

[2] Liu X , Wu J . History, applications, and challenges of immune repertoire research. Cell Biol Toxicol. 2018;34(6):441‐457. 29484527 10.1007/s10565-018-9426-0

[3] Rubelt F , Busse CE , Bukhari SAC , et al. Adaptive immune receptor repertoire community recommendations for sharing immune‐repertoire sequencing data. Nat Immunol. 2017;18(12):1274‐1278. 29144493 10.1038/ni.3873 PMC5790180

[4] Hou D , Chen C , Seely EJ , Chen S , Song Y . High‐throughput sequencing‐based immune repertoire study during infectious disease. Front Immunol. 2016;7:336. 27630639 10.3389/fimmu.2016.00336 PMC5005336

[5] Georgiou G , Ippolito GC , Beausang J , Busse CE , Wardemann H , Quake SR . The promise and challenge of high‐throughput sequencing of the antibody repertoire. Nat Biotechnol. 2014;32:158‐168. 24441474 10.1038/nbt.2782 PMC4113560

[6] Liu H , Pan W , Tang C , et al. The methods and advances of adaptive immune receptors repertoire sequencing. Theranostics. 2021;11(18):8945‐8963. 34522220 10.7150/thno.61390 PMC8419057

[7] Cabaniols JP , Fazilleau N , Casrouge A , Kourilsky P , Kanellopoulos JM . Most alpha/beta T cell receptor diversity is due to terminal deoxynucleotidyl transferase. J Exp Med. 2001;194(9):1385‐1390. 11696602 10.1084/jem.194.9.1385 PMC2195970

[8] Imkeller K , Wardemann H . Assessing human B cell repertoire diversity and convergence. Immunol Rev. 2018;284(1):51‐66. 29944762 10.1111/imr.12670

[9] Heather JM , Ismail M , Oakes T , Chain B . High‐throughput sequencing of the T‐cell receptor repertoire: pitfalls and opportunities. Brief Bioinform. 2018;19(4):554‐565. 28077404 10.1093/bib/bbw138 PMC6054146

[10] Rego SM , Snyder MP . High throughput sequencing and assessing disease risk. Cold Spring Harb Perspect Med. 2019;9(1):a026849. 29959131 10.1101/cshperspect.a026849 PMC6314070

[11] Parola C , Neumeier D , Reddy ST . Integrating high‐throughput screening and sequencing for monoclonal antibody discovery and engineering. Immunology. 2018;153(1):31‐41. 28898398 10.1111/imm.12838 PMC5721244

[12] Ma W , Yang Y , Zhu J , et al. Biomimetic nanoerythrosome‐coated aptamer‐DNA tetrahedron/maytansine conjugates: pH‐responsive and targeted cytotoxicity for HER2‐positive breast cancer. Adv Mater. 2022;e2109609. doi:10.1002/adma.202109609 35064993

[13] Li Y , Gao S , Shi S , et al. Tetrahedral framework nucleic acid‐based delivery of resveratrol alleviates insulin resistance: from innate to adaptive immunity. Nanomicro Lett. 2021;13(1):86. 34138319 10.1007/s40820-021-00614-6 PMC8006527

[14] Kocurova G , Ricny J , Ovsepian S . Autoantibodies targeting neuronal proteins as biomarkers for neurodegenerative diseases. Theranostics. 2022;12(7):3045‐3056. 35547759 10.7150/thno.72126 PMC9065204

[15] Zhang T , Tian T , Lin Y . Functionalizing framework nucleic‐acid‐based nanostructures for biomedical application. Adv Mater. 2021;e2107820. doi:10.1002/adma.202107820 34787933

[16] Ichinohe T , Miyama T , Kawase T , et al. Next‐generation immune repertoire sequencing as a clue to elucidate the landscape of immune modulation by host‐gut microbiome interactions. Front Immunol. 2018;9:668. 29666626 10.3389/fimmu.2018.00668 PMC5891584

[17] Crowe JE Jr . Influenza virus‐specific human antibody repertoire studies. J Immunol. 2019;202(2):368‐373. 30617118 10.4049/jimmunol.1801459 PMC6327975

[18] Xiao Z , Huang Q , Yang Y , et al. Emerging early diagnostic methods for acute kidny injury. Theranostics. 2022;12(6):2963‐2986. 35401836 10.7150/thno.71064 PMC8965497

[19] Zhou Q , Yan X , Zhu H , et al. Identification of three tumor antigens and immune subtypes for mRNA vaccine development in diffuse glioma. Theranostics. 2021;11(20):9775‐9790. 34815785 10.7150/thno.61677 PMC8581422

[20] Cole C , Volden R , Dharmadhikari S , Scelfo‐Dalbey C , Vollmers C . Highly accurate sequencing of full‐length immune repertoire amplicons using Tn5‐enabled and molecular identifier‐guided amplicon assembly. J Immunol. 2016;196(6):2902‐2907. 26856699 10.4049/jimmunol.1502563

[21] Tang C , He Z , Liu H , et al. Application of magnetic nanoparticles in nucleic acid detection. J Nanobiotechnol. 2020;18(1):62. 10.1186/s12951-020-00613-6 PMC7171821 32316985

[22] He Z , Tong Z , Tan B , et al. Rapid detection of DNA methylation with a novel real‐time fluorescence recombinase‐aided amplification assay. J Biomed Nanotechnol. 2021;17(7):1364‐1370. 34446139 10.1166/jbn.2021.3111

[23] Hou Y , Liu Y , Tang C , et al. Recent advance in nanomaterials for cancer immunotherapy. Chem Eng J. 2022;435(2):134145.

[24] Liu J , Yang X , Lu X , et al. Impact of T‐cell receptor and B‐cell receptor repertoire on the recurrence of early stage lung adenocarcinoma. Exp Cell Res. 2020;394(2):112134. 32540399 10.1016/j.yexcr.2020.112134

[25] Wang X , Hu Y , Liu X , et al. Quantitative characterization of T‐cell repertoire alteration in Chinese patients with B‐cell acute lymphocyte leukemia after CAR‐T therapy. Bone Marrow Transplant. 2019;54(12):2072‐2080. 31383996 10.1038/s41409-019-0625-y

[26] Miyasaka A , Yoshida Y , Wang T , Takikawa Y . Next‐generation sequencing analysis of the human T‐cell and B‐cell receptor repertoire diversity before and after hepatitis B vaccination. Hum Vaccin Immunother. 2019;15(11):2738‐2753. 30945971 10.1080/21645515.2019.1600987 PMC6930056

[27] Chang CM , Feng PH , Wu TH , Alachkar H , Lee KY , Chang WC . Profiling of T cell repertoire in SARS‐CoV‐2‐infected COVID‐19 patients between mild disease and pneumonia. J Clin Immunol. 2021;41(6):1131‐1145. 33950324 10.1007/s10875-021-01045-z PMC8096628

[28] Blackman MA , Woodland DL . The narrowing of the CD8 T cell repertoire in old age. Curr Opin Immunol. 2011;23(4):537‐542. 21652194 10.1016/j.coi.2011.05.005 PMC3163762

[29] Rettig TA , Tan JC , Nishiyama NC , Chapes SK , Pecaut MJ . An analysis of the effects of spaceflight and vaccination on antibody repertoire diversity. Immunohorizons. 2021;5(8):675‐686. 34433623 10.4049/immunohorizons.2100056 PMC10996920

[30] Fink K . Can we improve vaccine efficacy by targeting T and B cell repertoire convergence? Front Immunol. 2019;10:110. 30814993 10.3389/fimmu.2019.00110 PMC6381292

[31] Zhou M , Gao S , Zhang X , et al. The protective effect of tetrahedral framework nucleic acids on periodontium under inflammatory conditions. Bioact Mater. 2021;6(6):1676‐1688. 33313447 10.1016/j.bioactmat.2020.11.018 PMC7708773

[32] Ma W , Zhan Y , Zhang Y , Mao C , Xie X , Lin Y . The biological applications of DNA nanomaterials: current challenges and future directions. Signal Transduct Target Ther. 2021;6(1):351. 34620843 10.1038/s41392-021-00727-9 PMC8497566

[33] de Bourcy CF , Angel CJ , Vollmers C , et al. Phylogenetic analysis of the human antibody repertoire reveals quantitative signatures of immune senescence and aging. Proc Natl Acad Sci U S A. 2017;114(5):1105‐1110. 28096374 10.1073/pnas.1617959114 PMC5293037

[34] Sas K , Szabó E , Vécsei L . Mitochondria, oxidative stress and the kynurenine system, with a focus on ageing and neuroprotection. Molecules. 2018;23(1):191. 10.3390/molecules23010191 PMC6017505 29342113

[35] Niu X , Li S , Li P , et al. Longitudinal analysis of T and B cell receptor repertoire transcripts reveal dynamic immune response in COVID‐19 patients. Front Immunol. 2020;11:582010. 33117392 10.3389/fimmu.2020.582010 PMC7561365

[36] Yang Y , Wang C , Yang Q , et al. Distinct mechanisms define murine B cell lineage immunoglobulin heavy chain (IgH) repertoires. Elife. 2015;4:e09083. 26422511 10.7554/eLife.09083 PMC4714975

[37] Mogilenko DA , Shchukina I , Artyomov MN . Immune ageing at single‐cell resolution. Nat Rev Immunol. 2021;1‐15. doi:10.1038/s41577-021-00646-4 PMC8609266 34815556

[38] Moura J , Madureira P , Leal EC , Fonseca AC , Carvalho E . Immune aging in diabetes and its implications in wound healing. Clin Immunol. 2019;200:43‐54. 30735729 10.1016/j.clim.2019.02.002 PMC7322932

[39] Ramasamy MN , Minassian AM , Ewer KJ , et al. Safety and immunogenicity of ChAdOx1 nCoV‐19 vaccine administered in a prime‐boost regimen in young and old adults (COV002): a single‐blind, randomised, controlled, phase 2/3 trial. Lancet. 2021;396(10267):1979‐1993. 33220855 10.1016/S0140-6736(20)32466-1 PMC7674972

[40] Chen Y , Klein SL , Garibaldi BT , et al. Aging in COVID‐19: vulnerability, immunity and intervention. Ageing Res Rev. 2021;65:101205. 33137510 10.1016/j.arr.2020.101205 PMC7604159

[41] Li Z , Huang Z , Li X , et al. Bioinformatic analyses hinted at augmented T helper 17 cell differentiation and cytokine response as the central mechanism of COVID‐19‐associated Guillain‐Barré syndrome. Cell Prolif. 2021;54(5):e13024. 33751722 10.1111/cpr.13024 PMC8088459

[42] Zhang M , Zhang X , Tian T , et al. Anti‐inflammatory activity of curcumin‐loaded tetrahedral framework nucleic acids on acute gouty arthritis. Bioact Mater. 2021;8:368‐380. 34541407 10.1016/j.bioactmat.2021.06.003 PMC8429917

[43] Qin X , Xiao L , Li N , et al. Tetrahedral framework nucleic acids‐based delivery of microRNA‐155 inhibits choroidal neovascularization by regulating the polarization of macrophages. Bioact Mater. 2021;14:134‐144. 35310341 10.1016/j.bioactmat.2021.11.031 PMC8892086

[44] Lin T , Buffenstein R . The unusual immune system of the naked mole‐rat. Adv Exp Med Biol. 2021;1319:315‐327. 34424522 10.1007/978-3-030-65943-1_12

[45] Cunha LL , Perazzio SF , Azzi J , Cravedi P , Riella LV . Remodeling of the immune response with aging: immunosenescence and its potential impact on COVID‐19 immune response. Front Immunol. 2020;11:1748. 32849623 10.3389/fimmu.2020.01748 PMC7427491

[46] Thevarajan I , Nguyen THO , Koutsakos M , et al. Breadth of concomitant immune responses prior to patient recovery: a case report of non‐severe COVID‐19. Nat Med. 2020;26(4):453‐455. 32284614 10.1038/s41591-020-0819-2 PMC7095036

[47] Diao B , Wang C , Tan Y , et al. Reduction and functional exhaustion of T cells in patients with coronavirus disease 2019 (COVID‐19). Front Immunol. 2020;11:827. 32425950 10.3389/fimmu.2020.00827 PMC7205903

[48] Chen YT , Hsu HC , Lee YS , et al. Longitudinal high‐throughput sequencing of the T‐cell receptor repertoire reveals dynamic change and prognostic significance of peripheral blood TCR diversity in metastatic colorectal cancer during chemotherapy. Front Immunol. 2021;12:743448. 35095836 10.3389/fimmu.2021.743448 PMC8789675

[49] Lian J , Liu J , Yue Y , et al. The repertoire features of T cell receptor β‐chain of different age and gender groups in healthy Chinese individuals. Immunol Lett. 2019;208:44‐51. 30905825 10.1016/j.imlet.2019.03.007

[50] Peng W , Liu S , Meng J , et al. Profiling the TRB and IGH repertoire of patients with H5N6 avian influenza virus infection by high‐throughput sequencing. Sci Rep. 2019;9(1):7429. 31092835 10.1038/s41598-019-43648-y PMC6520366

[51] Heather JM , Best K , Oakes T , et al. Dynamic perturbations of the T‐cell receptor repertoire in chronic HIV infection and following antiretroviral therapy. Front Immunol. 2015;6:644. 26793190 10.3389/fimmu.2015.00644 PMC4707277

[52] Chen L , Zhao J , Peng J , et al. Detection of SARS‐CoV‐2 in saliva and characterization of oral symptoms in COVID‐19 patients. Cell Prolif. 2020;53(12):e12923. 33073910 10.1111/cpr.12923 PMC7645955

[53] He Z , Chen Z , Tan M , et al. A review on methods for diagnosis of breast cancer cells and tissues. Cell Prolif. 2020;53:e12822. 32530560 10.1111/cpr.12822 PMC7377933

[54] Xie W , Shen J , Wang D , et al. Dynamic changes of exhaustion features in T cells during oral carcinogenesis. Cell Prolif. 2022;55(4):e13207. 35179267 10.1111/cpr.13207 PMC9055910

[55] Chen J , Du L , Wang F , et al. Cellular and molecular atlas of the placenta from a COVID‐19 pregnant woman infected at midgestation highlights the defective impacts on foetal health. Cell Prolif. 2022;55(4):e13204. 35141964 10.1111/cpr.13204 PMC9055894

[56] Cai B , Gong Y , Wang Z , Wang L , Chen W . Microneedle arrays integrated with living organisms for smart biomedical applications. Theranostics. 2021;11(20):10012‐10029. 34815801 10.7150/thno.66478 PMC8581439

[57] Wang Z , Su G , Dai Z , et al. Circadian clock genes promote glioma progression by affecting tumour immune infiltration and tumour cell proliferation. Cell Prolif. 2021;54(3):e12988. 33442944 10.1111/cpr.12988 PMC7941241

[58] Song Y , Huang Y , Zhou F , Ding J , Zhou W . Macrophage‐targeted nanomedicine for chronic diseases immunotherapy. Chin Chem Lett. 2022;33(2):597‐612.

[59] Li J , Luo H , Zhu X , Zhao J , Chen T . Designing DNA cage‐based immuno‐fluorescence strategy for rapid diagnosis of clinical cervical cancer tissues. Chin Chem Lett. 2022;33(2):788‐792.

[60] Liu X , Zheng C , Kong Y , Wang H , Wang L . An in situ nanoparticle recombinant strategy for the enhancement of photothermal therapy. Chin Chem Lett. 2022;33(1):328‐333.

