# Supplementary material for: Immune repertoire analysis of normal Chinese donors at different ages
Source: Cell Prolif. 2022 Aug 4;55(11):e13311. doi: 10.1111/cpr.13311 (PMC9628227; doi:10.1111/cpr.13311)
Supplement: Supplementary file 1 — FIGURE S1 V‐J gene usage. (A) V‐J gene usage of TRA between the two groups. (B) V‐J gene usage of TRD between the two groups. (C) V‐J gene usage of TRG between the two groups. (D) V‐J gene usage of IGK between the two groups. (E) V‐J gene usage of IGL between the two groups. Different colored arcs in the outer circle represent V/J gene types, and the size of the arc represents the frequency. The area of the inner circle V‐J gene linkage represents the combined V‐J gene expression frequency. [file CPR-55-e13311-s001.docx]

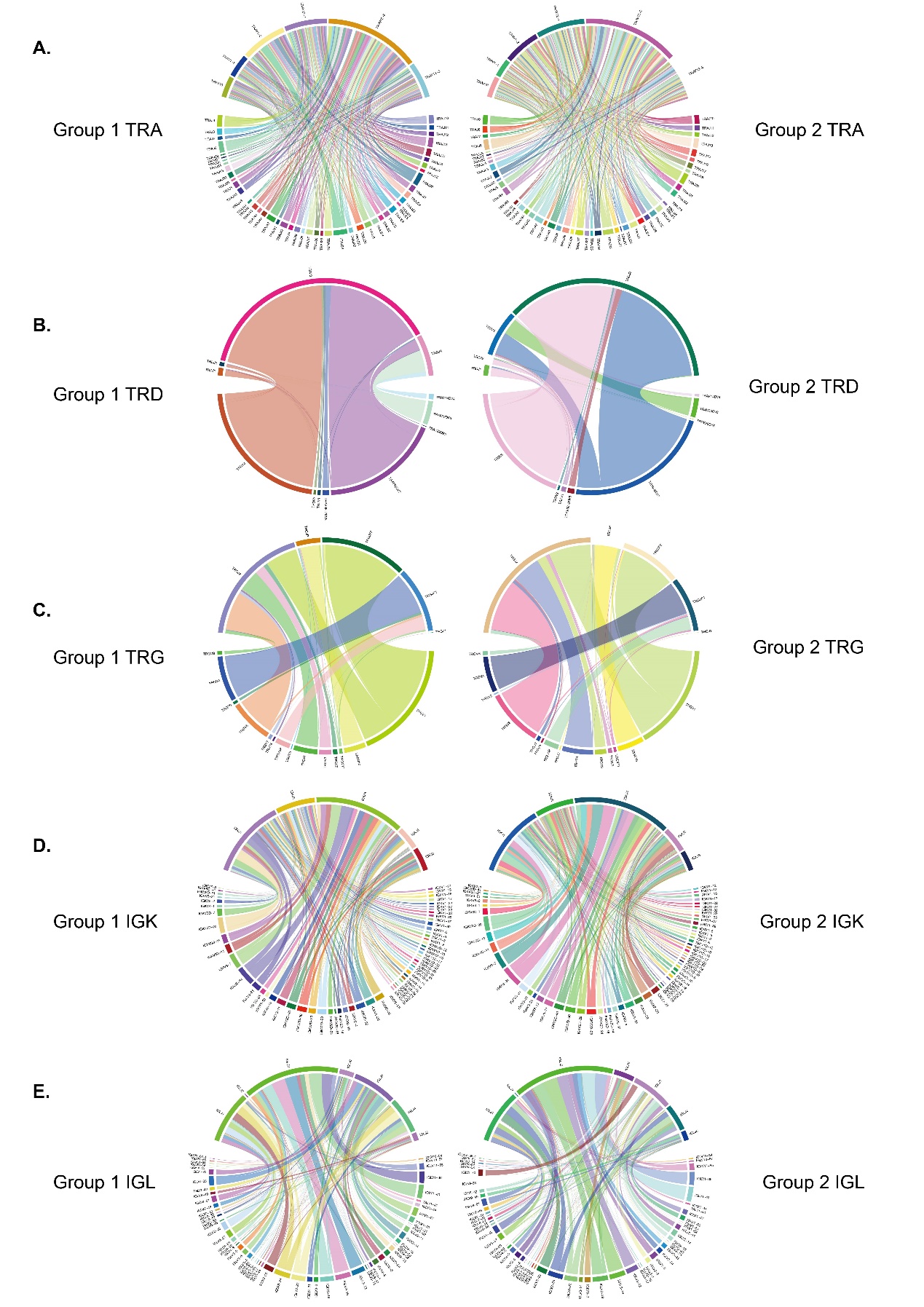


**Figure S1. V-J gene usage. (A) V-J gene usage of TRA between the two groups. (B) V-J gene usage of TRD between the two groups. (C) V-J gene usage of TRG between the two groups. (D) V-J gene usage of IGK between the two groups. (E) V-J gene usage of IGL between the two groups. Different colored arcs in the outer circle represent V/J gene types, and the size of the arc represents the frequency. The area of the inner circle V-J gene linkage represents the combined V-J gene expression frequency.**
